# Feasibility of Using Floor Vibration to Detect Human Falls

**DOI:** 10.3390/ijerph18010200

**Published:** 2020-12-29

**Authors:** Yu Shao, Xinyue Wang, Wenjie Song, Sobia Ilyas, Haibo Guo, Wen-Shao Chang

**Affiliations:** 1 School of Architecture, Harbin Institute of Technology, Harbin 150001, China; shaoyu@hit.edu.cn (Y.S.); 18S034002@stu.hit.edu.cn (X.W.); 18S034014@stu.hit.edu.cn (W.S.); 2 Key Laboratory of Cold Region Urban and Rural Human Settlement Environment Science, Ministry of Industry and Information Technology, Harbin 150001, China; 3 School of Architecture, The University of Sheffield, Sheffield S10 2TN, UK; silyas2@sheffield.ac.uk

**Keywords:** fall detection, floor vibrations, machine learning, elderly, health and wellbeing, intelligent system

## Abstract

With the increasing aging population in modern society, falls as well as fall-induced injuries in elderly people become one of the major public health problems. This study proposes a classification framework that uses floor vibrations to detect fall events as well as distinguish different fall postures. A scaled 3D-printed model with twelve fully adjustable joints that can simulate human body movement was built to generate human fall data. The mass proportion of a human body takes was carefully studied and was reflected in the model. Object drops, human falling tests were carried out and the vibration signature generated in the floor was recorded for analyses. Machine learning algorithms including K-means algorithm and K nearest neighbor algorithm were introduced in the classification process. Three classifiers (human walking versus human fall, human fall versus object drop, human falls from different postures) were developed in this study. Results showed that the three proposed classifiers can achieve the accuracy of 100, 85, and 91%. This paper developed a framework of using floor vibration to build the pattern recognition system in detecting human falls based on a machine learning approach.

## 1. Introduction

An aging population is causing issues in several countries and this is an increasing trend. By 2050, the old-age dependence ratio (the number of people who are 65 and over relative to those between 15 and 64) in the European Union is expected to double to 54% [1]. Aging can cause the decline in bone mineral density and physical function [2], which makes older people more vulnerable to accidents. Studies have shown that falls constitute one of the leading causes of severe injury in the elderly [3]. Almost 20% of community-dwelling older people experience accidental falls every year [4]. Two thirds of all severe injuries sustained on the elderly are caused by falls [5]. The situation becomes more serious when a senior adult is unable to stand up after a fall, especially when living alone [6]. Over half of those who remain on the ground for more than an hour after a fall experience a deterioration in general health, resulting in death within six months even when there is no direct injury from the fall. Older people living alone can easily fall without anyone noticing, and it can take considerable time for an alarm to be raised due to them being alone. They can remain on the floor for a prolonged time, with severe adverse consequences [7]. The development of automatic fall-detection methods to detect a fall and ensure that medical help arrives in time has become of increasing importance. Different fall postures can lead to different kinds of injury. Forward falls will lead to high potential of joint dislocations and upper limb injuries, while backward falls often lead to increased risk of head injuries [8]. It is essential that fall postures be identified by a fall detection system to ensure that action can be taken in time.

Since falls are often associated with injuries and possibly more serious consequences, several studies have been carried out to detect the occurrence of falls in the elderly in order to prevent them from remaining on the ground for too long. There are three mainstream methods for fall detection that are currently available and are being used in by older adults in different living settings.

### 1.1. Visual-Based Fall Detecting Systems

Visual-based fall detecting systems can monitor the elderly throughout surveillance cameras in real-time in a home care setting. Thermal vision cameras can also be applied in certain cases [9,10,11]. The criteria chosen for fall detection after subtraction can be further categorized into three branches, namely, time of inactivity, body movement, and posture recognition. The first approach is based on how long the person remains in a reclined position state after a period of inactivity by setting up an inactivity history map [12]. This method can generate false alarms when persons are resting. In the second approach, parameters like velocity of movement [13], displacement [12] and orientation of the head position [14], body centroid or spine [15] are used to evaluate whether there is a fall. However, this approach does not give good results when people are engaged in strenuous exercise [16]. In the posture recognition approach, various methods such as projection histograms of the segmented silhouette [17], pixel-based subtraction algorithms [18], Kalman filtering [19], and genetic algorithms [20] are applied to extract the human silhouette from the background in surveillance images. Several features such as the aspect ratio, orientation angle [14], and distance to the floor [21] are extracted to analyze shape changes so as to distinguish the fall event from daily life activities.

The installation of cameras in homes to monitor the elderly in real-time is undoubtedly an efficient method to detect falls with high accuracy. However, it is undeniable that this form of surveillance could make the elderly feel observed and infringe their privacy, which may lead to increased stress and resistance. This is not conducive to creating a safe and comfortable home environment for older people.

### 1.2. Wearable Sensor-Based Fall Detecting Systems

Another method of detecting falls in the elderly is through the use of wearable sensors. There are different approaches and different systems have different algorithms and therefore a different number of sensors can be used [22,23,24,25]. The use of pairs of sensors such as a combined accelerometer and gyroscope [26,27,28,29] or a combined accelerometer and barometer [30,31] constitute the two leading options in current research. These sensors can work together to collect various data including orientation in terms of yaw, pitch, and roll angles [32], frequency domain [33], and the acceleration of multiple body parts, especially the waist regions [34,35,36,37]. Data can be extracted from these sensors to classify different human activities, including falls. Wearable sensors can collect accurate data and give out signals in a timely fashion. However, this method does rely on the sensors being attached to the wearer, and if the person does not have the sensors attached, it is not possible to detect when the fall occurs.

### 1.3. Floor Vibration-Based Fall Detecting Systems

The vibration generated by a fall can provide a good approach to the development of a fall detection system. Alwan et al. proposed a fall detection system where a particular piezoelectric sensor is attached to the floor surface to collect the vibration signals, and a pre-processing circuit powered by batteries is applied to analyze the vibration patterns and transfer them as a binary signal in the case of a fall event [33]. Fall experiments were conducted by multiple participants falling on purpose in this research. This experimental method can be too subjective to fully simulate an unconscious fall. Alwan’s method was further developed by Werner into an automatic fall detection system focusing on appraising the practical feasibility of this method as well as further validation and development of the relative techniques. Werner used a dummy to simulate falls in this study [38]. These methods proved the feasibility of using floor vibration to determine a fall although the experiments did not focus on the way of falling. Yazar et al. demonstrated a fall detection system composed of a vibration sensor and two passive infrared sensors [39]. This approach increases accuracy but reduces fault tolerance. Liu et al. used a multi-features semi-supervised support vector machine algorithm to process and analyze the floor acceleration through installed accelerometers and managed to recognize human falls [40]. Liu’s team deliberately adopted falls in different postures for the related experiments, but this was only aimed at increasing the diversity of experimental samples rather than deliberately distinguishing between falls with different postures.

In comparison with visual-based detection systems and wearable sensors, the use of floor vibration to determine fall events not only ensures high accuracy with relatively low financial investment, but also manages to avoid placing any further physical or mental burden on users. However, there is a lack of current research into the floor vibration-based fall detecting system’s ability to distinguish falls with different postures.

Although there are several existing fall detecting systems, there is limited research examining fall posture identification. Most proposed systems tended to use high-sensitivity sensors, which are too expensive for widespread use in housing for the elderly. On the basis of current related studies, the aims of this research are as follows:

1. The feasibility of identifying falls from other activities based on floor vibration signals collected by high-sensitivity sensors has already been demonstrated. This work investigates whether fall detection using low-sensitivity mobile built-in sensors can achieve sufficiently high accuracy.
2. Are the results efficient enough to identify different fall postures based on floor vibration signals collected by mobile built-in sensors?

## 2. Method

This paper combines fall experiments with a machine learning approach to realize fall detection through the use of floor vibration signals.

### 2.1. Experiments

#### 2.1.1. Human Body Model Formulation

**Figure 1:**
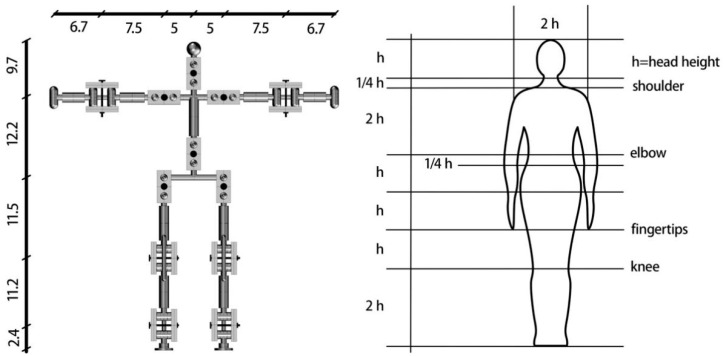
Proportion of body parts of the model (data source [41]).

**Figure 2:**
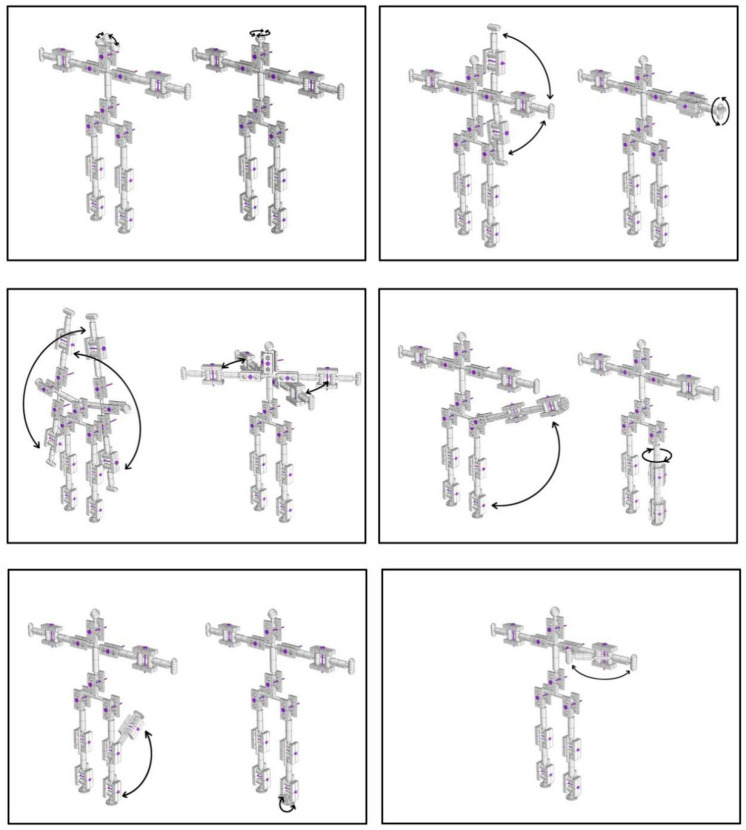
The moving mode and range of each joint in the model.

**Figure 3:**
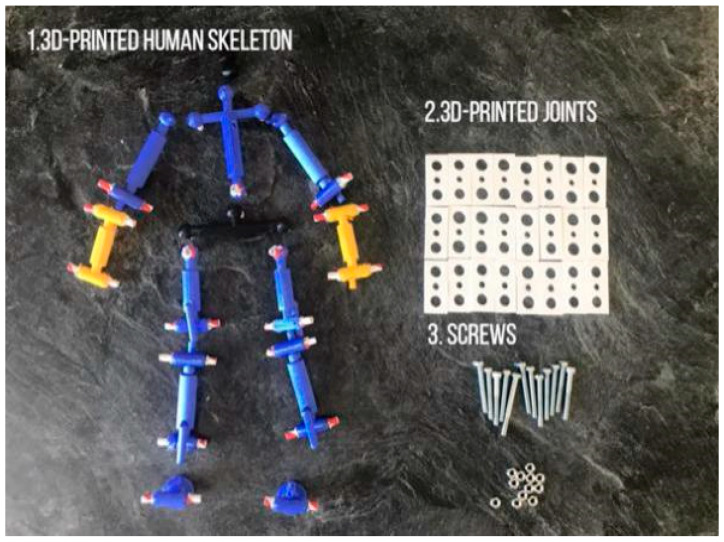
Components required for model assembly.

**Figure 4:**
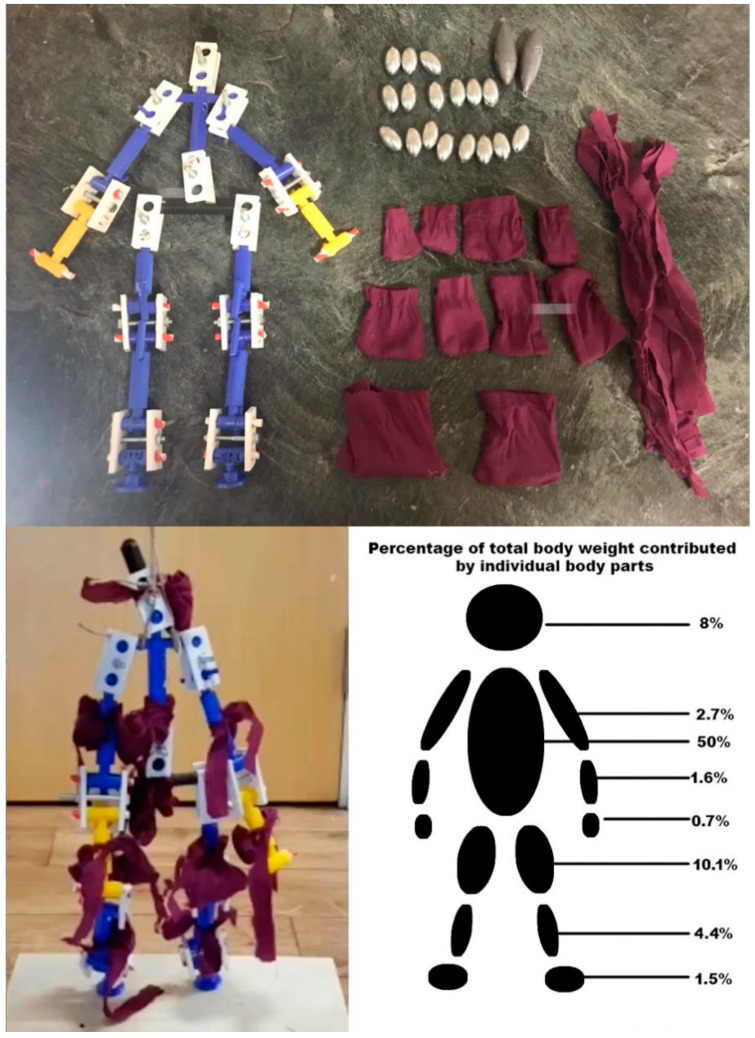
Model load (data source [42]).

In order to simulate the posture of a fall precisely, a physical model developed at a scale of 1:4 to actual human body size was fabricated. The ratio of the length of each body part in this model is consistent with the actual ratios of the human body, with reference to [41] (Figure 1), and the main joints connecting different body parts function in the same way as actual human joints in terms of the direction and range of movement (Figure 2). The model was 3D printed and the body parts were assembled with joints and screws to ensure mobility (Figure 3). The model is used to simulate a conscious person who is capable of controlling all of his limbs and standing up straight when all screws are tightened. It can simulate an unconscious person unable to control his body when all the model joints are loosened. The body weight proportions were studied and these are reflected in the model by attaching weights to the skeleton [42], as shown in Figure 4.

#### 2.1.2. Experimental Procedure

**Figure 5:**
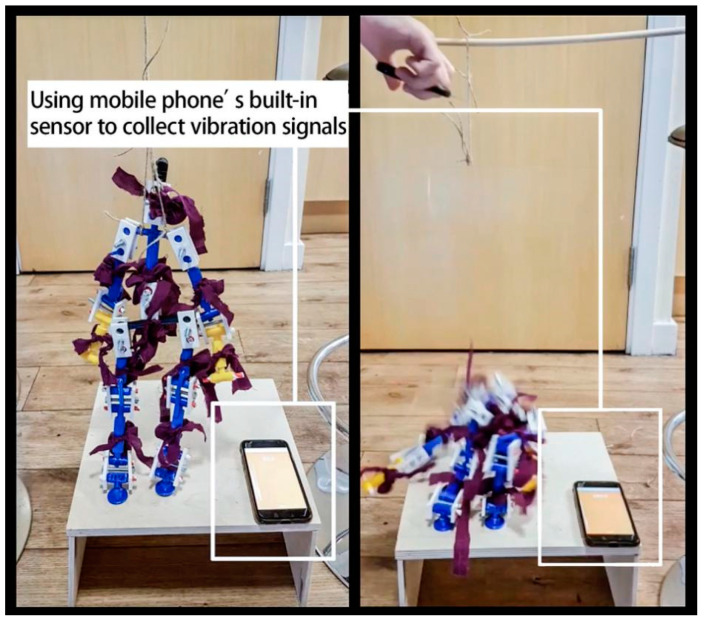
Experiment setup.

**Figure 6:**
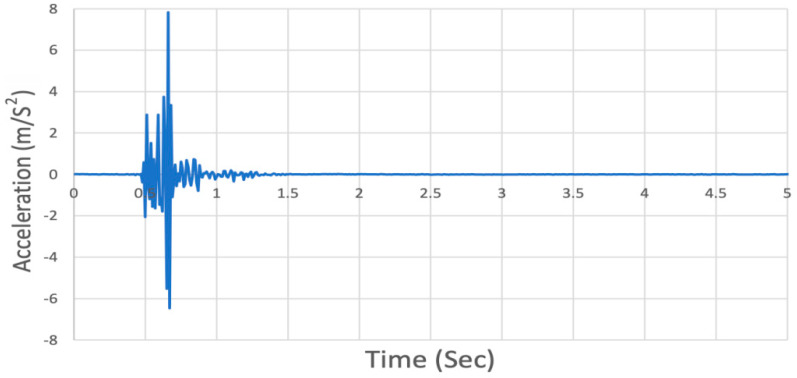
Time-history record for human fall.

The fall experiments are carried out on a 12 × 12-inch plywood sheet, and the vibration from the model’s falls are recorded. A mobile phone with a built-in accelerometer is used to collect the vibration data (Figure 5). The purpose was to utilize a low-sensitivity accelerometer instead of using a high-sensitivity accelerometer to detect human falls. The sample frequency of the test is 100 Hz. The acceleration chart obtained from the mobile phone is shown in Figure 6.

**Figure 7:**
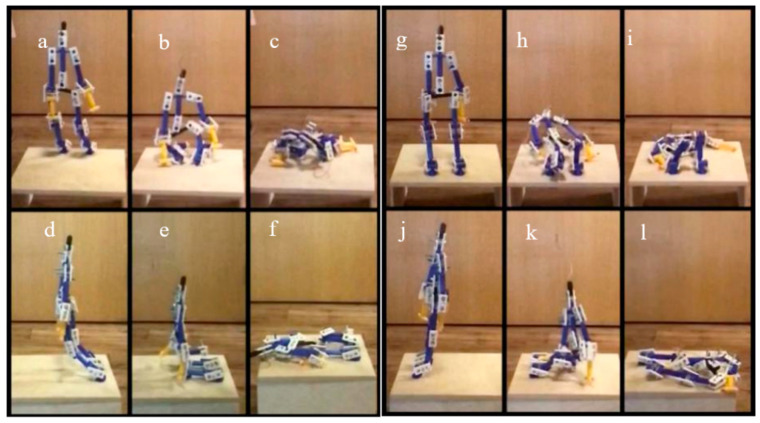
The process for forward and backward fall. (**a**) Front of the first step of forward fall; (**b**) Front of the second step of forward fall; (**c**) Front of the third step of forward fall; (**d**) The left side of the first step of forward fall; (**e**) The left side of the second step of forward fall; (**f**) The left side of the third step of forward fall; (**g**) Front of the first step of backwards fall; (**h**) Front of the second step of backwards fall; (**i**) Front of the third step of backwards fall; (**j**) The left side of the first step of backwards fall; (**k**) The left side of the second step of backwards fall; (**l**) The left side of the third step of backwards fall.

Figure 6 clearly shows that there is rapid attenuation after the fall, so only the first five seconds are used in this analysis. Three different activities, namely human forward fall, human backward fall, and object drop, are included in this study. In the human fall experiments, the model is placed in the center of the plywood board, with head and shoulder parts tied to a support above to keep the body up straight at the beginning. The posture changes during the human forward fall process are shown in Figure 7 using a sequence of images. Before the fall occurs, the model has all screws loosened to simulate the unconscious state, and the entire body is leant forward.

**Figure 8:**
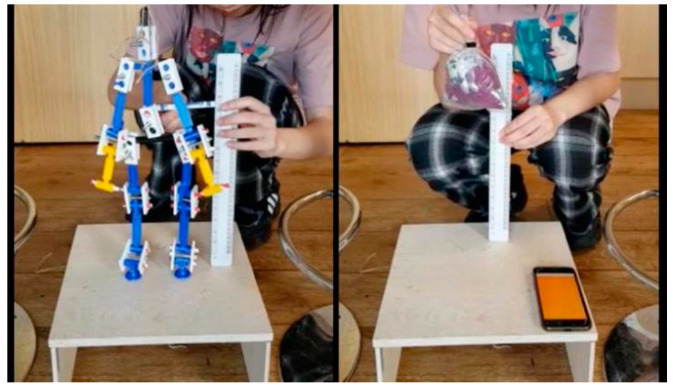
The experiment of object drops.

After releasing the rope holding the model, the knees land first as the body leans forward (Figure 7a,d), followed by the upper body and arms (Figure 7b,e), and the head lands last (Figure 7c,f)). In the case of the backward fall, the model’s initial state is also with all joints loosened while leaning backward. As the rope is released, as shown in Figure 8, the entire body leans leaning backward with the knees bent and the hips landing first (Figure 7h,k). After that, the upper body continues to fall back due to inertia, with the upper limbs and the head touching the floor at the end (Figure 7i,l).

A series of object drop tests were also carried out (Figure 8). In the object drop experiment, the total weight of the test object is the same as the human model and the object is dropped from the same height as the center of gravity of the model. The vibration data is recorded from the time of the fall to the end of the last rebound. The purpose of the object fall test is to ensure whether human falls can be distinguished from other one-drop activities by assessing the floor vibration data.

A total of 314 experiments were conducted during the entire process, consisting of 107 object drops, 97 sets of human forward falls, and 110 sets of human backward falls.

### 2.2. Proposed Fall Detection Algorithms

#### 2.2.1. K-Means Clustering Algorithm

In this paper, the K-means clustering method is introduced to modify the raw data at the outset. The K-means clustering algorithm is an iterative solution clustering analysis algorithm. The method’s aim is to divide an N-dimensional population into K sets on the basis of a sample with reasonably efficient partitions in the sense of within-class variance [43].

The standard algorithm for K-means clustering consists of two parts [44]. The first step is the initial assignment, where K objects are selected randomly as initial cluster centers(centroids), and then each observation is assigned to the cluster with the least squared Euclidean distance. Given a set of observations (*X*_1_, *X*_2_, …, *X_n_*), where each observation is a m-dimensional real vector, K-means clustering aims to partition the *n* observations into *k* (≤*n*) sets *C* = {*C*_1_, *C*_2_, …, *C_k_*}. Equation (1) is a mathematical representation that can be used to describe the initial assignment:

(1) $$dis\left(X_{i},C_{j}\right)=\sqrt{\overset{m}{\underset{t=1}{\sum }}{\left(X_{it}-C_{jt}\right)}^{2}}\;\;1\leq i\leq n,\;1\leq j\leq k,\;1\leq t\leq m$$

where *X_is_* is the *ith* observation, *C_j_* is the *jth* cluster center, $X_{it}$ is the *tth* vector of the *ith* observation, and $C_{jt}$ is the *tth* vector of the *jth* cluster center

The next step is to recalculate centroids for observations assigned to every cluster and reassign each observation into a new cluster. This process is repeated until a certain termination condition is met. The termination condition is that no (or a minimum number of) objects are reassigned to different clusters, no (or minimum number) of centroids change again, and the sum of squared errors is also minimum. Equation (2) can be used to describe the recalculation:

(2) $$C_{l}=\frac{\sum_{X_{i}\in S_{l}}X_{i}}{\left|S_{l}\right|}\;1\leq l\leq k,\;1\leq i\leq \left|S_{l}\right|$$

where *C_l_* is the *lth* cluster’s centroid, *|S_l_|* is the number of the observation in the *lth* cluster, and *X_i_* is the *ith* observation in the *lth* cluster

Since K is pre-set according to different data sets, the value that can best reflect the characteristics of the pattern is different when processing different data patterns. One way to find the most appropriate *k* value in cluster analysis is the elbow method, which is a heuristic method to determine the number of clusters in a data set. The elbow method uses different values of *k* to plot the value of the mean absolute deviation of the dataset [45]. As the value of *k* increases, the mean absolute deviation also increases, and the instances approach the centerline of the corresponding graph. However, the increase in the mean absolute deviation decreases as the value of *k* increases. The *k* value at the steepest drop point in the dispersion is called the elbow, where diminishing returns are no longer worth the additional cost of further clusters. Given a set {*x*_1_, *x*_2_, …, *x_n_*}, Equation (3) can be used to calculate the mean absolute deviation for the elbow method:

(3) $$M_{D}=\frac{1}{n}\overset{n}{\underset{i=1}{\sum }}\left|x_{i}-m\left(X\right)\right|\;1\leq i\leq n$$

where *M_D_* is the mean absolute deviation, *x_i_* is the *ith* observation, and *X_i_* is the *ith* observation in the given set, *m*(*X*) is the mean value for the given set

#### 2.2.2. K-Nearest Neighbor Algorithm

The K-nearest neighbor algorithm is a non-parametric method proposed by Thomas Cover used for classification and regression [46]. It is a relatively mature method and one of the simplest machine learning algorithms. The main concept of this method is that if most of the K numbers for the nearest samples of a chosen sample in the featured space belong to a certain category, the chosen sample also belongs to the same category and has the characteristics of the samples in this category.

### 2.3. Pre-Defined Values in the Algorithms

In the process of developing the binary classification system, the value of specific parameters in the algorithm needs to be pre-defined before the system is performed. The pre-defined values include the total time of extracted data (T), the K value in the K-means clustering algorithm (K_1_), and the K value in the K-nearest neighbor algorithm (K_2_).

**Figure 9:**
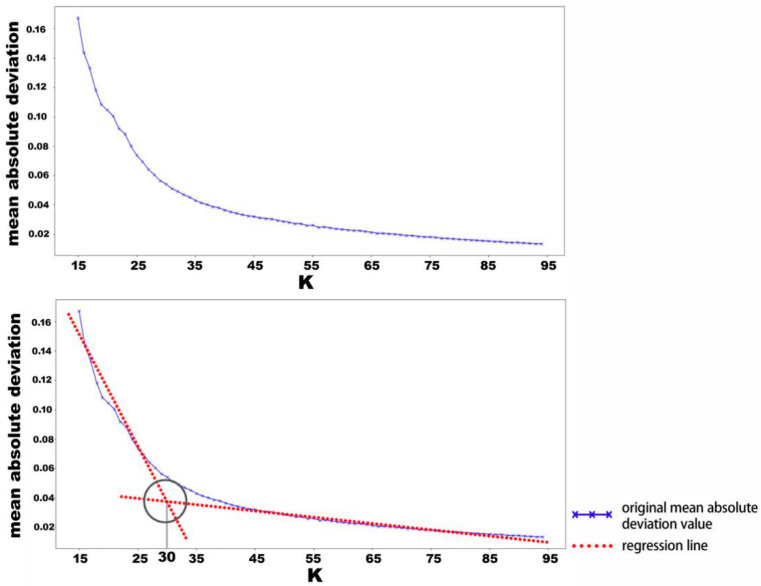
Selection of the K value using the elbow method.

**Figure 10:**
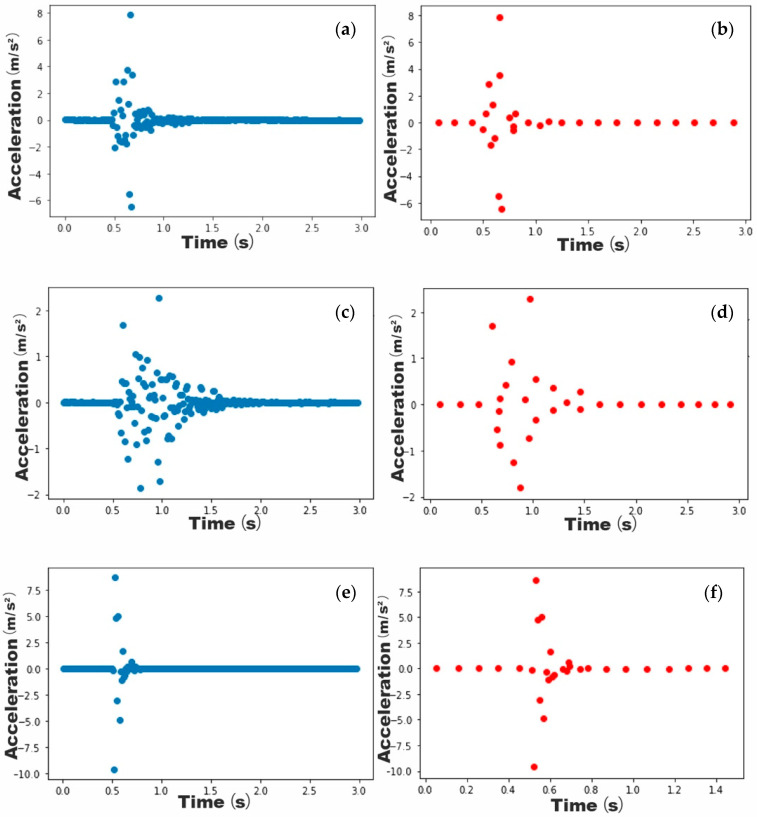
Pattern generation when K = 30. (**a**) Original pattern of human forward fall; (**b**) Simplified pattern of human forward fall; (**c**) Original pattern of human backwards fall; (**d**) Simplified pattern of human backwards fall; (**e**) Original pattern of object drop; (**f**) Simplified pattern of object drop.

By clustering similar data using the K-means algorithm, the continuous pattern obtained from the experiments can be processed into discrete data. Taking a set of the vibration signals collected from a human fall as an example, the mean absolute deviation of the data is shown in Figure 9. Since there is no obvious steep drop point in the graph, the intersection point of the regression lines at the beginning and end of the graph is regarded as the yield point when using the elbow method to determine the most reasonable number of clusters in the dataset. Using the elbow algorithm, the optimal value of K_1_ is determined to be 30. The examples of the patterns generated for the three different experimental activities after adopting K-means clustering using 30 as the K value are shown in Figure 10.

Since the total length of time for each pattern is 5 s, the time before the activity starts is 0.5 s and the longest vibration time in a pattern is 1 s for all the data sets, the value of T can range from 1.5 to 5 s. Theoretically speaking, the smaller T is, the more pronounced the pattern features can be. However, if the value of T is too small, the classifier’s fault tolerance rate will also decrease accordingly. The value of K_2_ should be a single positive integer. The control variates method can be used to determine the values of both pre-determined parameters for the three classifications.

Table 1 shows the performance of the classifier with different T values under the same condition, while Table 2 shows the accuracy with different k_2_ values. The most reasonable values can be selected by comparison. These are T = 3 for people walking and falling with different falling postures, T = 1.5 for people falling and object drops, while K = 7.

## 3. Results and Analysis

### 3.1. Human Walking versus Human Fall

In order to ensure that the algorithm can distinguish a human walking from a fall, a series of walking signals were generated. The signals were generated using the software OpenSees and the process was discussed in [47].

**Figure 11:**
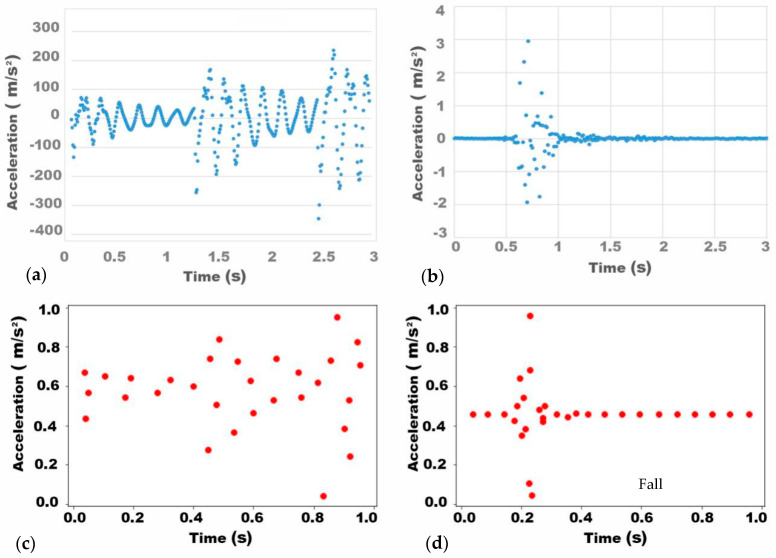
Standardized patterns for walking and fall. (**a**) original walking pattern; (**b**) original fall pattern; (**c**) simplified walking pattern; (**d**) simplified fall pattern.

The ratio of the standard pattern for walking generated by the simulation software is different from the fall pattern generated through the experiments, and the values in both patterns need to be scaled to a range of 0–1 for future comparison. Figure 11 shows an example of the two sets of data after normalization.

Since walking is a continuous behavior, the floor acceleration over time during the entire activity is widely distributed. In contrast, a fall is a single-drop behavior, and the timber floor starts free decay after the fall (Figure 11). Hence, the patterns for these two different human activities are significantly different in terms of:

① the length of the vibration (parameter 1)
② the amount of data within the vibration (parameter 2)

Table 3 shows clear differences between the two parameters in terms of mean value, maximum and minimum values, and standard deviation for the different behavior. Based on this observation, the two activities can be distinguished by setting these parameters as criteria.

Cross-validation and the K-nearest neighbor algorithm were applied to train the binary classifier. In order to use the K-nearest neighbor algorithm to train the classifier, all the vibration data were divided into five random groups. Each group was then taken as the test set while the remaining groups are taken in turn as the training set. Table 4 shows the performance of the classifier using each parameter in five simulations when taking K = 7, with good results. Parameter 1 gave better results with accuracy, precision, and recall of 100%.

### 3.2. Human Fall Versus Object Drop

**Figure 12:**
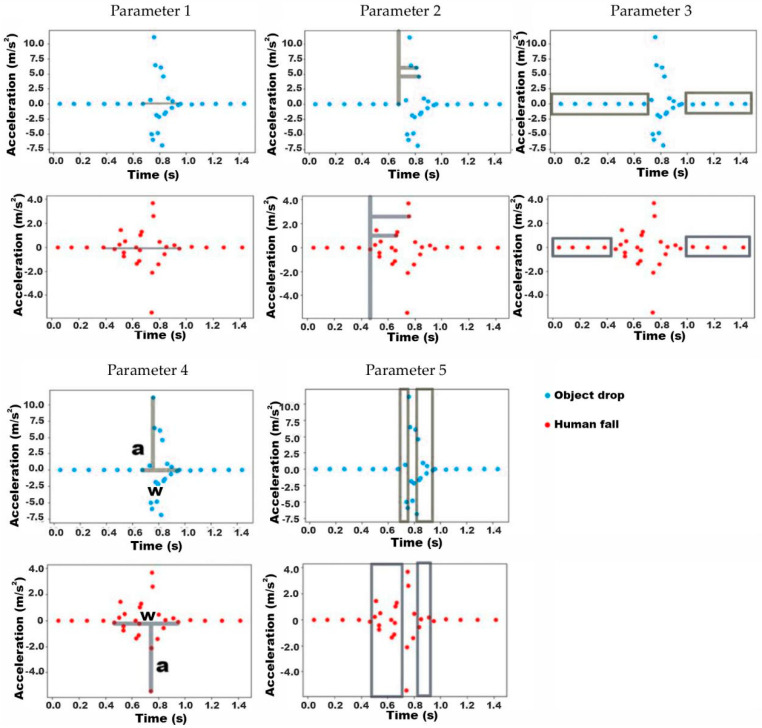
Extracted parameters of human fall and object drop.

Unlike the binary classification of human walking and human fall, human fall and object drop are both one-drop activities, which means the total length of vibration in these two activities is relatively short in both cases. Therefore, when distinguishing between the patterns for the two activities, it is necessary to focus on the data distribution within the vibration section period. In an object drop, the object touches the ground first and bounces back several times until it stops. These activities cause the floor to vibrate multiple times, but the amplitude of each vibration gradually decreases over time. However, in case of a human fall, although the floor also generates multiple vibrations since body parts can touch floor synchronously, the sequence of body parts touching the ground is uncertain, and hence the maximum acceleration of these vibrations may not decrease over time. It can be seen from Figure 12 that the peak point appeared relatively later and the total vibration duration was comparatively longer in the human fall pattern. This means that there is less vibration data located near 0 on the y-axis in the human fall pattern in comparison to the object drop pattern. In terms of the distribution of the data during vibration, the data from the human fall pattern are relatively widely scattered while the data from the object drop pattern generally appear around the same time as the wave peak. Since the weight of the object and of the human model are the same, the maximum instantaneous floor acceleration is greater for the object drop than for the human fall.

**Figure 13:**
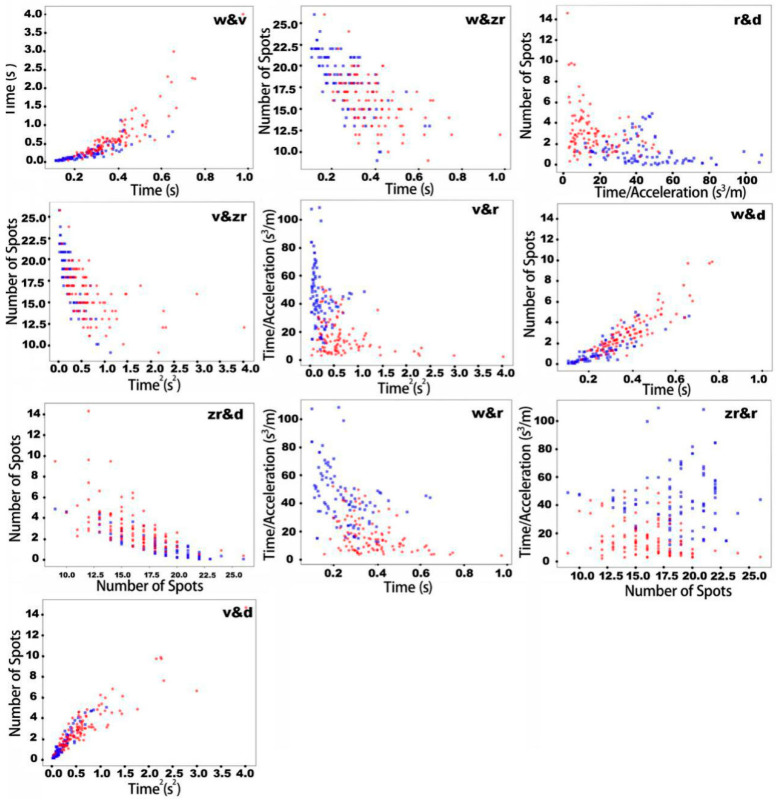
Plot of human fall (red dots) and object drop (blue dots) with different combined parameters (parameter 1: w, parameter 2: v, parameter 3: zr, parameter 4: r, parameter 5: d).

In order to digitize these features, assuming the total number of data points in the vibration is *n*, the names of the data points are p_1_, p_2_, p_3_, …, p_n_, the coordinates are (x_1_, y_1_), (x_2_, y_2_), (x_3_, y_3_) …. (x_n_, y_n_), and the extracted parameters are listed as follows (Figure 12):

① The total length of the vibration: w = x_n_ − x_1_
② The dispersion of data in vibration: v = (x_2_ − x_1_)^2^ + (x_3_ − x_1_)^2^ + (x_4_ − x_1_)^2^ + … + (x_n_ − x_1_)^2^
③ The number of data points located outside the vibration section (zr)
④ The aspect ratio of the vibration section: r = w/a, where w = x_n_ − x_1_, a = max (|y_n_|) (Figure 13, Parameter 4)
⑤ The number of data points within the vibration which do not appear at the same time as the peak (d)

Table 5 shows the average, maximum and minimum, and standard deviation for these five parameters from the human fall and object drop datasets. These values vary between the two activities, but the difference is not pronounced.

Cross-validation and the K-nearest neighbor algorithm were again applied to train the binary classifier. The performance of the classifier using each parameter as the criterion for the five is shown in Table 6. It can be seen that accuracy, precision, and recall for all parameters were poor in this case, with values below 75%.

In order to increase accuracy, instead of using only one parameter as a criterion, a combination of two parameters was applied to improve the classifier. In the plots in Figure 13, the x-axis and y-axis of the graphs respectively represent the value of one parameter. Red dots on the plots represent human fall cases while the blue ones represent object drop cases. The results from combining ten parameters in pairs are shown in Figure 13. Table 7 shows the performance of the classifier using these five combinations of parameters as criteria. With these new criteria, accuracy increases significantly, with three sets of combinations reaching an accuracy of over 80%, and the best performance overall achieved by r&d with an accuracy of 85%. The values for precision and recall for each parameter also increased significantly.

### 3.3. Human Falls from Different Postures

**Figure 14:**
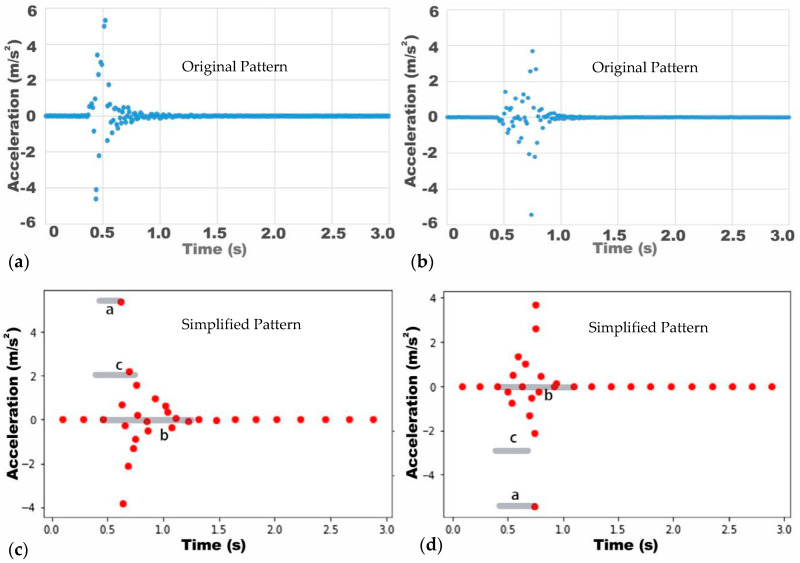
Features extracted from pattern for forward and backward falls. (**a**) Original Pattern of backwards fall; (**b**) Original Pattern of forward fall; (**c**) Simplified Pattern of backwards fall; (**d**) Simplified Pattern of forward fall.

Two types of fall postures are examined in this work, the forward and the backward fall. When a person falls forward, the knee touches the ground first, followed by the upper body. When a person falls backward, the hip often touches the ground first (Figure 8). In terms of human body weight distribution (Figure 5), the heaviest part is the upper body, which accounts for about half of the total weight. Therefore, when a person leans forward and falls, the upper body touches the ground later, and the position of the relative wave crest in the corresponding pattern should be visible during the second half of the vibration. When a person falls backward with the hips touching the ground first, the first wave crest should be the highest wave crest of the entire pattern, and this appears relatively early. Based on this theoretical hypothesis, assuming the total time of the vibration is b, the time difference between the start of the vibration and the appearance of the peak point is a, and the distance between the centroid of all data points inside the vibration section and the first data point inside the vibration section along the x-axis is c (Figure 14). The extracted parameters are listed as follows:

① The ratio of the time between the appearance of the peak and the start of the vibration to the total time of the vibration: port = a/b
② The ratio of the distance between the centroid of all data points within the vibration and the vibration’s starting point to the total length of the vibration along the x-axis: ave = c/b
③ The ratio of the distance between the centroid of all data points within the vibration and the vibration’s starting point to the distance between the vibration’s starting point and the peak point along the x-axis: sca = a/c

Table 8 shows the performance of these parameters when used to distinguish different fall postures. It can be seen that the maximum, minimum, and average values for the three values differ for the different datasets. Therefore, it is reliable to use these parameters as criteria to recognize human fall postures.

When K = 7 in the K nearest neighbor algorithm, the accuracy, precision, and recall using a single parameter and the combined parameters as the classification criteria are shown in Table 9. The accuracy can exceed 0.9 when using the combined parameters port&ave as the criterion. The value of precision and recall both increase accordingly.

### 3.4. The Framework for Detection

**Figure 15:**
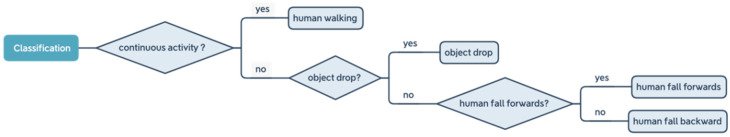
Work flow for the classification system.

A flowchart is established in this work to address the procedure for detecting human falls (Figure 15). For starters, the system needs to distinguish single drop activities from continuous activities. Here, human walking is taken as representative of continuous activity, with two parameters selected as the criteria to achieve 100% accuracy in distinguishing between these two activities. After recognizing single drop activities, the next step is to distinguish human falls from object drops. Five parameters are extracted from the patterns as criteria. Since the single parameter is not sufficiently accurate to distinguish activities, a combination of parameters was applied to improve the classifier. With a combination of parameter r and parameter d as the criterion, accuracy of classification reaches 85%. After recognizing human falls, the final classifier to distinguish different fall postures was proposed. Three parameters are extracted to determine the fall posture. An accuracy of 91% can be achieved by using a combination of two parameters as the criterion.

## 4. Discussion

(1) Using a 3D-printed model to simulate unconscious fall.

In former studies, researchers have collected floor vibration data for different falls by asking participants to fall on purpose. It is hard to simulate unconscious falls using this method as the participants are controlling the sequence of the landing body parts involuntarily even when attempting not to. Using a human body model to simulate falls can avoid this problem and this method can achieve high accuracy. Liu and his team used a dummy to simulate falls from a standing position [40], while Alwan and his team used two dummies to simulate falls from standing position and sitting position, respectively [33]. Both of the proposed classifiers achieved high accuracy. The results from this research demonstrated that a one-to-four scaled 3D-printed model can also achieve high accuracy when compared to related research outcome. This model is formulated to simulate unconscious falls under different postures by controlling the body hinges. This can expand the current field of study and provides a new approach to analyzing human activity for future studies.

(2) Using simplified patterns of certain activities in feature extraction and classification.

Instead of analyzing the physical characteristics of the raw data, the simplified patterns of certain activities are obtained before classification in this study. Features were extracted directly from the obtained patterns. A machine learning approach was introduced in this step to preprocess vibration data. Clustering not only reduced the total amount of data and accelerated the procedure, but also made the data features more eminent and easier to extract in the further classification. Pattern recognition in classification has already been successfully applied to various fields, such as computer-aided diagnosis, machine vision, data mining, and knowledge discovery. However, behavior recognition based on floor vibration is still in its initial stages. The pretreating of data provides a new approach in human activity recognition.

(3) Feasibility of using mobile built-in sensors to detect falls.

The study explored the feasibility of using mobile built-in sensors to detect falls, which can be further used for wireless networking between hospital and older peoples’ homes. Unlike formerly developed fall detecting systems using high-sensitivity sensors, the proposed method can achieve high accuracy even with low-sensitivity sensors. Compared with installing sensors in older people’s home, which is expensive and difficult to promote, using mobile phones to detect human fall is more affordable and accessible. Since the proposed system relied on a mobile’s built-in sensor, the collected data can be transmitted in real time to the cloud. For further development, an app could be developed based on this method to satisfy the demand for wireless networking between hospital and older peoples’ home in the context of the upcoming 5G era. This could help the hospital to prepare and treat patient in the shortest duration possible. Apart from in housing for older people, this application can also be widely adopted in hospitals and nursing homes to reduce the risk to older residents and enhance the working efficiency of staff.

(4) The significance in identifying fall postures.

Many researchers have already succeeded in using floor vibration to identify falls from other activities. However, there is limited research examining floor vibration to recognize fall postures, even though the way people fall has a significant influence on the health outcomes. There are three common postures in most falls, namely forwards, backward, and sideways [48]. Different fall postures have different levels of injurious consequences [49]. This research focused on the situation of unconscious falls, and since sideways falls do not often occur in unconscious falls, the work here only considers forward and backward falls. Forward falls are always accompanied by soft tissue injuries, joint dislocations, and upper limb injuries. The high potential energy of a fall and a hard landing surface are known to be independent risk factors for hip fractures when a sideways fall occurs [8]. The backward fall has an increased risk of head injury, which can lead to severe long-term sequelae. If older people do not get the right medical treatment in time after a fall, their health situation will deteriorate quickly. Therefore, having a detecting system which can identify fall posture after a fall and apprise the hospital about the patient’s situation in advance can help with the medical process. The proposed system can achieve 91% accuracy using mobile phone to identify fall postures.

## 5. Conclusions

The purpose of this study is to develop an intelligent detection system embedded within a building to determine when an unconscious fall occurs and to distinguish between the different fall postures based on the floor vibration data. The system improves safety in the home environment and enhance health care for the elderly.

To realize this, this paper investigates the use of machine learning algorithms as classification methods to identify fall events with different postures. By applying the K-means algorithm and the K-nearest neighbor algorithm, the fall detection system successfully classifies the patterns generated by the various fall activities through the application of machine learning.

The performance of the proposed method is validated experimentally, with two variations of simulated human falls using a 3D-printed model with adjustable joints as well as object drops. Three classifiers were developed to distinguish human falls from human walking, human falls from object drops, and human forward falls from human backward falls. The results showed that the accuracy of fall identification in these three classifications reached 100, 85, and 91%, respectively. In summary, the results confirmed the performance of the proposed system and demonstrated great potential in distinguishing falls from other activities, as well as identifying the different fall postures from the floor vibration. The classification system developed in this research proved the feasibility of this novel method using algorithms in machine learning to build a pattern recognition system to detect human falls.

The system developed achieved high accuracy in identifying falls and in recognizing fall postures. This can expedite medical treatment as hospitals can be pre-informed about the nature of the fall experience by elderly patients. Moreover, the proposed system is based on low-sensitivity mobile built-in sensors, which are accessible and affordable. It can be widely promoted and installed in older peoples’ apartments and nursing homes to improve the life quality of the elderly without interrupting their normal daily routine.

## Figures and Tables

**Table 1:** Accuracy of classification with different T values.

Parameter Setting	Binary Classification of People Walking and Falling	Binary Classification of People Falling and Object Drops	Binary Classification of Different Falling Postures
T =1.5 s, K_1_ = 30, K_2_ = 9	0.82	0.82	0.85
T = 2 s, K_1_ = 30, K_2_ = 9	0.87	0.78	0.85
T = 2.5 s, K_1_ = 30, K_2_ = 9	0.85	0.77	0.86
T = 3 s, K_1_ = 30, K_2_ = 9	0.89	0.78	0.87
T = 3.5 s, K_1_ = 30, K_2_ = 9	0.88	0.81	0.83
T = 4 s, K_1_ = 30, K_2_ = 9	0.78	0.80	0.80

**Table 2:** Accuracy of classification with different K_2_ values.

Parameter Setting	Binary Classification of People Walking and Falling (T = 3)	Binary Classification of People Falling and Object Drops (T = 1.5)	Binary Classification of Different Falling Postures (T = 3)
K_1_ = 30, K_2_ = 3	0.89	0.79	0.86
K_1_ = 30, K_2_ = 5	0.91	0.79	0.87
K_1_ = 30, K_2_ = 7	0.92	0.82	0.88
K_1_ = 30, K_2_ = 9	0.89	0.82	0.87

**Table 3:** Values of the selected parameters in different data sets.

Parameter	Value	Human Walking	Human Fall
Parameter 1: Vibration Time (second)	Average value	0.5267	0.3856
	Maximum value	0.5875	0.9757
	Minimum value	0.4344	0.1674
	Standard deviation	0.0459	0.1334
Parameter 2: Number of dots	Average value	28.7142	13.9800
	Maximum value	30	20
	Minimum value	27	4
	Standard deviation	1.2777	2.8305

**Table 4:** Index of the classification using a single parameter.

Parameter	Index	Cross Validation Iteration 1	Cross Validation Iteration 2	Cross Validation Iteration 3	Cross Validation Iteration 4	Cross Validation Iteration 5	Average Value
Parameter 1	accuracy	1	1	1	1	1	1
	precision	1	1	1	1	1	1
	recall	1	1	1	1	1	1
Parameter 2	accuracy	1	0.98	1	1	1	0.996
	precision	1	0.875	1	1	1	0.975
	recall	1	0.93	1	1	1	0.986

**Table 5:** Performance of selected parameters in different data sets.

Parameter	Value	Human Fall	Object Drop
Parameter 1: w (second)	Average value	0.3856	0.2576
	Maximum value	0.9756	0.6501
	Minimum value	0.1674	0.1094
	Standard deviation	0.1333	0.1104
Parameter 2: v (second ^2^)	Average value	0.6800	0.2336
	Maximum value	4.0155	1.1266
	Minimum value	0.0348	0.0140
	Standard deviation	0.6174	0.2178
Parameter 3: zr (number of dots)	Average value	16.0238	18.5000
	Maximum value	26	26
	Minimum value	10	9
	Standard deviation	2.8241	3.4071
Parameter 4: r ($\frac{second^{3}}{m}$)	Average value	15.9050	42.0024
	Maximum value	52.0430	108.7313
	Minimum value	2.3360	7.4762
	Standard deviation	12.0696	20.3400
Parameter 5: d (number of dots)	Average value	3.1042	1.2543
	Maximum value	14.6349	4.9231
	Minimum value	0.3348	0
	Standard deviation	2.1492	1.2223

**Table 6:** Index of classification of human falls and object drops.

Parameter	Index	Cross Validation Iteration 1	Cross Validation Iteration 2	Cross Validation Iteration 3	Cross Validation Iteration 4	Cross Validation Iteration 5	Average Value
Parameter 1 (w)	Accuracy	0.563	0.637	0.594	0.627	0.548	0.594
	Precision	0.688	0.563	0.688	0.706	0.684	0.6658
	Recall	0.688	0.563	0.688	0.75	0.812	0.7002
Parameter 2 (v)	Accuracy	0.75	0.656	0.656	0.688	0.718	0.694
	Precision	0.722	0.692	0.667	0.688	0.684	0.6906
	Recall	0.813	0.563	0.625	0.688	0.812	0.7002
Parameter 3 (zr)	Accuracy	0.75	0.625	0.625	0.656	0.593	0.645
	Precision	0.786	1	1	0.857	0.667	0.862
	Recall	0.688	0.25	0.25	0.375	0.375	0.3876
Parameter 4 (r)	Accuracy	0.755	0.7	0.737	0.775	0.775	0.7484
	Precision	1	0.786	1	0.909	1	0.939
	Recall	0.688	0.688	0.625	0.625	0.687	0.6626
Parameter 5 (d)	Accuracy	0.594	0.625	0.557	0.594	0.6	0.594
	Precision	0.722	0.588	0.667	0.684	0.684	0.669
	Recall	0.813	0.625	0.625	0.813	0.813	0.7378

**Table 7:** Index of the classification of human fall and object drop with combined parameters.

Parameter Combination	Index	Cross Validation Iteration 1	Cross Validation Iteration 2	Cross Validation Iteration 3	Cross Validation Iteration 4	Cross Validation Iteration 5	Average Value
w&v	Accuracy	0.75	0.813	0.75	0.75	0.75	0.7626
	Precision	0.722	0.778	0.75	0.72	0.722	0.7384
	Recall	0.813	0.875	0.75	0.812	0.812	0.8124
w&zr	Accuracy	0.758	0.779	0.792	0.715	0.775	0.7638
	Precision	0.716	1	0.705	0.736	0.733	0.778
	Recall	0.75	0.688	0.75	0.875	0.687	0.75
w&r	Accuracy	0.754	0.775	0.797	0.713	0.775	0.7628
	Precision	1	0.785	1	0.909	1	0.9388
	Recall	0.688	0.687	0.625	0.625	0.688	0.6626
w&d	Accuracy	0.75	0.718	0.718	0.718	0.75	0.7308
	Precision	0.7	0.705	0.705	0.684	0.7	0.6988
	Recall	0.875	0.75	0.75	0.812	0.875	0.8124
v&zr	Accuracy	0.844	0.853	0.846	0.862	0.843	0.8496
	Precision	0.824	0.736	0.778	0.823	1	0.8322
	Recall	0.875	0.875	0.875	0.875	0.688	0.8376
v&r	Accuracy	0.815	0.835	0.785	0.85	0.835	0.824
	Precision	1	0.909	1	0.909	1	0.9636
	Recall	0.688	0.625	0.625	0.625	0.688	0.6502
v&d	Accuracy	0.688	0.812	0.781	0.687	0.718	0.7372
	Precision	0.667	0.812	0.909	0.687	0.684	0.7518
	Recall	0.75	0.812	0.625	0.687	0.812	0.7372
zr&r	Accuracy	0.784	0.784	0.784	0.767	0.778	0.7794
	Precision	0.923	0.812	0.923	0.833	0.786	0.8554
	Recall	0.75	0.812	0.75	0.625	0.688	0.725
zr&d	Accuracy	0.75	0.781	0.75	0.75	0.75	0.7562
	Precision	0.722	0.909	0.722	0.722	0.722	0.7594
	Recall	0.813	0.625	0.815	0.815	0.812	0.776
r&d	Accuracy	0.844	0.887	0.847	0.833	0.852	0.8526
	Precision	1	0.75	0.909	0.916	1	0.915
	Recall	0.688	0.562	0.625	0.687	0.625	0.6374

**Table 8:** Performance of selected parameters in different datasets.

Parameter	Value	Human Forward Fall	Human Backward Fall
Parameter 1: port (second)	Average value	0.6122	0.3579
	Maximum value	1	0.5511
	Minimum value	0.3549	0.1148
	Standard deviation	0.1295	0.1061
Parameter 2: ave (second)	Average value	0.5391	0.4604
	Maximum value	0.7731	0.6062
	Minimum value	0.3250	0.2739
	Standard deviation	0.0826	0.0834
Parameter 3: sca (second)	Average value	1.1367	0.7710
	Maximum value	1.5913	1.0841
	Minimum value	0.7575	0.2974
	Standard deviation	0.1664	0.1624

**Table 9:** Index of the classification of human fall postures with single and combined parameters.

Parameter Combination	Index	Cross Validation Iteration 1	Cross Validation Iteration 2	Cross Validation Iteration n3	Cross Validation Iteration 4	Cross Validation Iteration 5	Average Value
port	Accuracy	0.753	0.737	0.792	0.715	0.778	0.755
	Precision	0.706	0.706	0.7	0.9	0.722	0.7468
	Recall	0.8	0.8	0.8	0.6	0.815	0.763
ave	Accuracy	0.678	0.694	0.648	0.715	0.733	0.6936
	Precision	0.632	0.875	0.6	1	0.887	0.7988
	Recall	0.8	0.933	0.8	0.667	1	0.84
sca	Accuracy	0.9	0.889	0.878	0.92	0.9	0.8974
	Precision	0.875	0.825	0.875	1	0.667	0.8484
	Recall	0.933	0.767	0.93	0.667	1	0.8594
port&ave	Accuracy	0.9	0.937	0.894	0.9	0.915	0.9092
	Precision	0.875	1	0.825	0.722	0.684	0.8212
	Recall	0.93	0.688	0.767	0.815	0.812	0.8024
port&sca	Accuracy	0.8	0.834	0.745	0.836	0.815	0.806
	Precision	0.875	0.875	0.737	1	0.75	0.8474
	Recall	0.93	0.93	0.933	0.688	1	0.8962
ave&sca	Accuracy	0.912	0.878	0.885	0.925	0.947	0.9094
	Precision	0.737	0.825	0.875	1	0.736	0.8346
	Recall	0.933	0.767	0.93	0.688	0.933	0.8502
